# Dietary intake practices associated with cardiovascular risk in urban and rural Ecuadorian adolescents: a cross-sectional study

**DOI:** 10.1186/1471-2458-14-939

**Published:** 2014-09-09

**Authors:** Angélica Ochoa-Avilés, Roosmarijn Verstraeten, Carl Lachat, Susana Andrade, John Van Camp, Silvana Donoso, Patrick Kolsteren

**Affiliations:** Food Nutrition and Health Program, Universidad de Cuenca, Avenida 12 de Abril y Avenida Loja, Cuenca, Ecuador; Department of Food Safety and Food Quality, Faculty of Bioscience Engineering, Ghent University, Coupure Links 653, 9000 Gent, Belgium; Nutrition and Child Health Unit, Institute of Tropical Medicine, Nationalestraat 155, 2000 Antwerp, Belgium

**Keywords:** Diet, Adolescents, Cardiovascular risk, Socioeconomic status

## Abstract

**Background:**

Cardiovascular diseases (CVD) are amongst the leading causes of death worldwide. Risk factors of CVD develop during childhood and adolescence, and dietary quality has been linked to the development of CVD itself. This study examines the association between dietary patterns and cardiovascular risk in a group of urban and rural Ecuadorian adolescents from different socioeconomic backgrounds.

**Methods:**

A cross-sectional study was conducted from January 2008 to April 2009 among 606 adolescents from the 8th, 9th and 10th grade in an urban area (Cuenca), and 173 adolescents from a rural area (Nabón) in Ecuador. Data collection involved measuring anthropometric data (weight, height and waist circumference), blood pressure, dietary intake (2-day 24 h recall) and socio-demographic characteristics. Fasting blood lipids and glucose were measured in a subsample of 334 adolescents. Factor analysis was used to identify dietary patterns and linear regression models were used to (i) identify differences in food intake practices according to socioeconomic status and place of residence and (ii) establish relationships between dietary patterns and cardiovascular risk factors.

**Results:**

Median energy intake was 1851 kcal/day. Overall, fiber, fish and fruit and vegetables were scarcely consumed, while added sugar, refined cereals and processed food were important constituents of the diet. Two dietary patterns emerged, one labelled as “rice-rich non-animal fat pattern” and the other one as “wheat-dense animal-fat pattern”. The first pattern was correlated with a moderate increase in glucose in urban participants, while the second pattern was associated with higher LDL and cholesterol blood levels in rural participants.

**Conclusions:**

This group of adolescents presented various dietary practices conducive to CVD development. Effective strategies are needed to prevent CVD in the Ecuadorian population by encouraging a balanced diet, which contains less refined cereals, added sugar, and processed food, but has more fruits, vegetables and whole grain cereals.

## Background

Cardiovascular diseases (CVD) are the leading cause of death worldwide. This health problem is apparent in low-and middle-income countries (LMICs) [1] where CVD mortality currently exceeds that of infectious diseases [2]. Socioeconomic conditions play an important role in the risk of CVD, with the most disadvantaged populations being more at risk [3]. Currently, there is lack of information on the distribution of CVD in LMICs and on risk factors of other non-communicable diseases (NCD) with regard to socioeconomic status [3].

Identifying and understanding the distribution of risk factors is key to developing effective population intervention programs that aim to prevent CVD [4, 5]. Dietary quality has been associated as a possible risk factor with the development of CVD. Fresh fruit, vegetables, whole grains and fish consumption have been identified as important dietary factors protecting against CVD. Meanwhile a high intake of added sugar, sodium and other refined carbohydrates has been negatively associated with CVD in adults [6–9]. Nevertheless, an integral investigation of dietary behaviour and the link between diet and cardiovascular risk factors has not yet been assessed among adolescents from different socioeconomic backgrounds in LMICs. Available data principally originates from high-income countries [10, 11], and either takes a few aspects of dietary intake into consideration or focuses on a specific population group such as overweight adolescents [12, 13]. We have previously reported a high prevalence of CVD risk factors such as dyslipidemia, overweight and abdominal obesity in an Ecuadorian adolescent population with substantial differences between rural and urban areas [14].

This study examines the association between dietary characteristics and cardiovascular risk in a group of urban and rural Ecuadorian adolescents from different socioeconomic backgrounds. The findings of this study were used to design a culturally-acceptable school-based health-promoting intervention.

## Methods

### Participants

From January 2008 to April 2009 a cross-sectional study was conducted in a cluster random sample of adolescents in an urban area (n = 606), whilst all students from 8^th^, 9^th^ and 10^th^ grade willing to participate in the rural area were included (n = 173). A mean energy intake of 1700 kcal/d among school-going Ecuadorian children (E. Segarra, unpublished data, 2006) was used for sample size calculations. A precision of 10% and a cluster effect of 2 were taken into account for this. Anticipating possible not response, the original sample size was increased by14%. In total, 779 adolescents, aged 10–16 years old, from an urban (Cuenca) and rural (Nabón) area in Ecuador participated. Blood samples were collected in a subsample of 334 volunteers. A more detailed methodology of this study has been previously published [14].

The study protocol was approved by both the Ecuadorian and Belgian Ethical Committees (Nr: CBM/cobi-001 - 2008/462). An additional protocol for biochemical assessment was approved by the Ghent University Hospital Ethical Committee (Nr 2008100–97). Written consents were obtained from participants and their parents or guardians. Adolescents that were pregnant, had a concomitant disease, or were following a special diet were excluded from the study.

### Dietary assessment

Food intake was measured using an interview-administered 2-day 24-hour dietary recall. Days were randomly allocated to include one recall on a weekday and a second one on a weekend day per each participant [15]. Locally used utensils were calibrated and used to estimate portion sizes in order to quantify the amounts of food consumed. If the participants did not supply detailed information on the ingredients used and/or cooking methods of a recipe, recipes were prepared in triple by local volunteering housewives. For each of these recipes the ingredients and their weights were measured. The average was calculated which served as the final estimate for the ingredients and their weight of the recipe. In the case of uncommon recipes, such as some desserts or traditional dishes, an experienced cook was asked to prepare the recipe. The ingredients and their weights were recorded and calculated from this.

Since an up-to-date Ecuadorian food composition database does not exist, a compiled food composition database was developed using a pre-determined procedure. During the first stage all the 24-hour dietary recall forms were revised to make a list of all consumed recipes or ingredients. The second stage involved constructing a database using the following procedure: we searched the U.S. (USDA, 2012) database, when food items were not available the Mexican database (INNSZ, 1999) was used. If data could not be obtained, the Central American (INCAP/OPS, 2012) and Peruvian (CENAN/INS, 2008) databases were searched to compose the final food composition database. For locally processed and pre-packed food items, food labels were used to obtain the composition. A total of 13 food items were not available in any of the searched databases and data were obtained from analysis in our lab [16].

### Nutrients and recommendations

Added sugar was estimated by including all sugars used in any type of processed or prepared food (recipes), sugar naturally present in food was excluded from the analysis [8]. From a total of 556 identified food items, sugar content was available for 408 of them; either from the USDA or the Mexican food composition databases. For the remaining 148 food items, sugar content was estimated as follows: 58 food items were coded as containing natural sugar, since they were fruit, vegetables, grains, tubers or maize. For the remaining food items, either the sugar content of food items with similar nutritional characteristics was extrapolated (n = 35 food items), food labels were used (n = 42 food items), or the information of standardized recipes was used to quantify the sugar content (n = 12 food items). The sugar content of one food item (“Chicha de Jora”) could not be determined and was excluded from the analysis as it was consumed by only one participant. The following sources of added sugar were identified as described by Wesh et al.: Sweets (candies and gums, soda, added sugar and syrups, fruit flavoured drinks, pre-sweetened coffees and tea, sport drinks and energy drinks), grains (cake and cookies, ready to eat cereals, bread and muffins and other grains), fruits and vegetables, dairy (dairy desserts, milk, yogurt, and other dairy), protein sources (meat, egg and beans) and fat and oils [8].

Macronutrients and added sugar energy percentages per day (E%/day) were calculated by dividing the energy of each variable by the total energy intake per day. Macronutrient E% and sodium intake were compared with Dietary Reference Intakes (DRIs) for sex and age [17]. Since a clear definition of recommended added sugar intake is not available, we used the US recommendation of consuming <15% E% intake of solid fats and added sugar [18]. Therefore the variable solid fat was generated as described by the U.S. Department of Agriculture: all excess fat from the milk and meat and beans and solid fats added to foods in preparation or at the table, including cream, butter, stick margarine, regular or low-fat cream cheese, lard, meat drippings, cocoa, and chocolate [19]. The energy of added sugar and solid fats was summed up, expressed as E%/day and compared with the threshold of 15%. Total fruit and vegetable consumption were compared with the 400 g/d recommendation of the World Health Organization [20].

### Food groups

The food groups used in this study were based on the classification as proposed by the Health Department of Mexico [21] since this classification is in concordance with the objectives of this study and both the ingredients and recipes were comparable to those in Ecuador. After adapting this classification we identified a total of 20 main food groups: (i) white rice, (ii) refined wheat (bread, pasta, wheat powder), (iii) other refined cereals (tapioca, maize powder, banana powder and any other kind of powder different from wheat), (iv) whole grain cereals (quinoa, oat, barley), (v) maize, (vi) tubers, (vii) plantain, (viii) legumes, (ix) total fruit (including raw fruit and fruit used in juices or any other preparation), (x) vegetables, (xi) poultry, (xii) red meat (including processed meat), (xiii) fish and seafood, (xvi) dairy products (milk, yogurt, flavored milk, cheese), (xv) oilseeds (nuts, peanuts, almonds), (xvi) vegetable oils, (xvii) animal fat (butter, mayonnaise, crackling), (xviii) coffee, (xix) spices and (xx) processed food rich in salt, fat or added sugar included the following subgroups based on the Mexican classification which comprised (a) table sugar and sweets (honey, candies, chocolates, ice creams, sweet cookies, traditional sweet desserts and sugar added to juices, coffee, etc.), (b) salty snacks and fast food (all packaged salty snacks, salty cookies, French fries, pizza, hamburgers) (c) soft drinks (soda, artificial sweetened juices, energy drinks) and (d) any other packaged food (ketchup, packaged soups, gelatine). Food groups are presented as E%/day.

Food groups considered as protective (fruit, vegetables, oilseeds and fish) [7, 9] against CVD were further analysed in subgroups. As the type of fish could be important in determining this protective effect [22], we divided the reported fish consumption by processing methods, i.e. (i) fresh fish including steamed and roasted fish, (ii) fried fish and (iii) canned fish. As preparation methods may play a role in nutritional value [23], the most common preparation methods of fruit, vegetable, and oilseeds were also identified.

### Mealtimes

Mealtimes were defined as: breakfast, morning refreshment, lunch, afternoon refreshment, dinner and evening refreshment. The schools in this study have morning and afternoon schedules. In general, morning schools have classes from 7:00 until 13:00 and afternoon schools from 12:00 to 18:00. Mealtimes during the week were defined in accordance to these school hours. For morning schools the times were set as follows: breakfast between 5:00–7:00, morning snack between 7:00–13:00, lunch from 13:00–16:00 and afternoon snack from 16:00–18:00. For afternoon schools the timings were breakfast from 5:00–8:00, morning snack from 8:00–11:00, lunch from 11:00–12:00, afternoon snack from 12:00–18:00. Dinner and night snack were set equally for the whole sample from 18:00–21:00 and any hour later than 21:00 respectively. The weekend’s timing was set equally for all the participants. They were as follows: breakfast from 5:00–9:00, morning refreshment from 9:00–12:00, lunch from 12:00–15:00, and afternoon refreshment from 15:00–18:00.

### Cardiovascular risk factors

Body weight, height and waist circumference were measured in duplicate by trained researchers using calibrated equipment. Body mass index (BMI) was calculated as weight/height^2^ (kg/m^2^). Systolic blood pressure (SBP) and diastolic blood pressure (DBP) were measured on-site by trained staff in triplicate after a 10-min seated rest using a portable sphygmomanometer. A fourth measurement was taken if initial values were above 120/80 mmHg.

Blood samples were collected after overnight fasting. Blood serum was used to determine the total lipid profile. Total cholesterol (COLT), triglycerides (TG), and high-density lipoprotein cholesterol (HDL) were quantified using standardized methods [14]. For Low-density lipoprotein cholesterol (LDL) calculation the Friedewald formula [24] was used. Precision and accuracy were estimated: the global analytical coefficient of variation for COLT and TG was 3.1% and 5.7%, respectively. The % bias ranged from 0% to 8%.

### Socioeconomic status

Sociodemographic characteristics were assessed using the tool developed by the Integrated Social Indicator System for Ecuador [25]. This system defines poverty based on Unsatisfied Basic Needs (UBN). A household is considered as poor if one or more deficiencies in access to education, health, housing, urban services and employment are reported. Therefore adolescents were allocated to one of two groups; “Poor group” if at least one deprivation was present or “Better-off group” if no deprivation was reported.

### Data analysis

Food intake data was entered using an online software designed to analyse 24-hour recall data (Lucille software 0.1, 2010, Gent University; http://www.foodscience.ugent.be/nutriFOODchem/foodintake). Anthropometric, socioeconomic and blood lipid data were entered in duplicate into Epidata by two independent researchers. Any discrepancy was corrected using the original forms. Data are reported as mean with standard deviation (SD) or as median with 25–75 percentiles. When appropriate, continuous variables were transformed into a normal distribution. A significance level of 5% was determined for all statistical tests. Energy intake of macronutrients and food groups was adjusted for total energy intake using the nutrient residual model [26].

Factor analysis was carried out to identify dietary patterns including twenty food groups expressed as E%/day. The number of factors retained was based on a scree-plot. Food groups with a loading factor below 0.10 were removed from the analysis. Foods with a factor loading above 0.3 were identified as the main contributors to each pattern [27]. A score of each dietary pattern was calculated for each participant and split into tertiles. For each tertile of the dietary pattern score, the median of the macronutrients E%, added sugar and sodium contribution were reported as well as the mean of the cardiovascular risk factors.

Linear regression models were used with two purposes: (i) to identify differences in energy intake, macronutrient E%, added sugar, sodium and food group energy sources, by UBN and place of residence (ii) to compare, macronutrients E%, added sugar, sodium and cardiovascular risk factors across the tertiles of the dietary patterns score. The comparisons of dietary components and cardiovascular risk factors among the dietary patterns score tertiles were split into subgroups (UBN or place of residence) only if interactions were significant. Outcomes not following a normal distribution were log transformed before inclusion in the models, and beta coefficients were back transformed and expressed as percentage differences (estimate-1*100). All the models were adjusted for sex, UBN and place of residence when necessary.

Logistic regression models were used to determine UBN and place of residence differences in: (i) the amount of participants exceeding the macronutrient, sodium and added sugar recommended intake per day, (ii) the number of consumers of protective groups (subgroups) such as fish (roasted, fresh, canned) and fruit (preparation methods) as well as in (iii) the number of consumers of the main sources of added sugar. All the models were adjusted for sex, UBN and place of residence. Results are reported as odds ratio (OR) and 95% confidence interval (CI).

## Results

### Participants

No differences were found between the whole sample and the subsample of adolescents providing blood samples with regard to mean age (*P* = 0.24), BMI (*P* = 0.21), waist circumference (*P* = 0.52), systolic blood pressure (*P* = 0.72), diastolic blood pressure (*P* = 0.98), and sex (*P* = 0.19). Boys represented 50.1% of the total sample, yet more girls from the rural area participated in the study (*P* < 0.01). Mean age was 13.6 ± 1.2 (±SD) years and did not differ between sex, UBN or place of residence. Participants’ age ranged from 10–16 years old. Based on the UBN, 44% of the adolescents were classified as poor and a higher proportion of rural adolescents were poor compared with their urban peers (95% vs. 45% *P* < 0.01). Mean BMI was 20.3 ± 3.1 kg/m^2^ and girls had a higher BMI than boys (*P* < 0.01).

### Diet

In total 43 participants out of 779 provided only one recall, while for the remaining participants two days of dietary intake recall were available (one for a weekday and one for a weekend day). Energy intake, macronutrients E% and sodium intake are presented in Table 1. Median energy intake was 1851 kcal/day. Adolescents from the urban (8.0% less energy, *P < 0.01)* and the better off group (3.2% less energy, *P = 0.03)* got less energy from total carbohydrates when compared to their rural peers and the group classified as poor, respectively. This lower carbohydrate intake is due to a lower intake of carbohydrates other than added sugar (*P < 0.01 for both).* In contrast, added sugar was consumed more by adolescents from the higher socioeconomic strata (*P < 0.01).* Fat and protein were also highly consumed by urban adolescents as well as by those in the better off group.

**Table 1:** **Macronutrient, sodium and added sugar intake per day (median 25** ^**th**^ **-75** ^**th**^ **) according to UBN and place of residence**

Nutrients	Overall (n = 779)		UBN ^a^						Place of residence					
			Poor		Better off		β%	***P***	Rural		Urban		β***%***	***P***
Energy (kcal)	1851	[1507;2217]	1837	[1495;2161]	1857	[1528;2272]	0.1	0.95	1766	[1465;2076]	1863	[1528;2251]	4.3	0.08
Carbohydrates (E%)^b^	60.7	[55.7;64.9]	62.3	[57.7;65.9]	58.4	[53.9;62.9]	−3.2	0.01	63.8	[61.0;68.0]	59.2	[54.7;63.5]	−8.0	<0.01
Other CH (E%)	44.4	[38.8;50.8]	47.1	[42.0;52.9]	40.7	[35.4;46.3]	−8.7	<0.01	51.0	[46.9;55.4]	42.2	[36.9;47.8]	−14.9	<0.01
Added Sugar (E%)	15.4	[12.0;19.2]	14.4	[11.1;17.8]	16.5	[13.4;21]	15.4	<0.01	13.4	[9.9;16.4]	15.9	[12.8;19.9]	14.6	0.05
Fiber (g)	11.1	[8.0;16.0]	11.1	[8.2;16.3]	10.9	[7.9;15.2]	−1.8	0.63	12.3	[8.7;17.9]	10.8	[7.9;15.2]	−13.7	<0.01
Total Fat (E%)	24.2	[21.3;28.2]	23.1	[20.4;26.7]	25.8	[22.4;30.1]	5.2	<0.01	21.8	[18.6;24.3]	25.0	[21.9;29.1]	16.0	<0.01
Protein (E%)	13.4	[11.7;15.3]	13.0	[11.4;14.6]	14.2	[12.2;15.8]	4.2	0.04	12.0	[10.6;13.4]	13.8	[12.1;15.6]	11.5	<0.01
Sodium (mg)	2228	[1733;2907]	2099	[1672;2709]	2360	[1811;3087]	6.0	0.05	2038	[1596;2575]	2284	[1777;2991]	8.0	0.01

^a^Unsatisfied basic needs. ^b^Energy percentage. Differences using linear regression models.

The median intake of added sugar was 86.5 g (25^th^-75^th^, 60.8-120.9 g); its main sources were refined sugar added to fresh fruit-juices, coffee, tea, milkshakes (median intake 33.2 grams; 25^th^-75^th^, 22.4-45.0 g), sodas (median intake 15 grams; 25^th^-75^th^, 0–24.4 g), and bread and muffins (median intake 3.6 grams; 25^th^-75^th^, 3.0-6.2 g). Fiber intake was low among the whole sample. Urban adolescents ate less fiber than their rural pairs (*P < 0.01).*

### Recommendations

An estimated 23.9% of the participants exceeded the recommended E%/day of carbohydrates intake. Rural and poor adolescents were 3.1 *(P < 0.01; 95% CI, 1.6-5.7)* and 1.7 *(P = 0.02; 95% CI, 1.1-2.8)* times more likely to exceed their carbohydrate E%/day, respectively*.* In total, 92.1% of the adolescents got more than 15% of their daily energy intake from solid fats and added sugar, (median sugar intake = 15.4%). Adolescents in the better off group were almost three times as more likely to exceed this threshold *(P < 0.01; 95% CI, 1.4-6.0)*. Only 5.0% of the adolescents reached the recommended fiber intake with no statistical differences in UBN or place of residence.

A small number of participants (5.7%) exceeded the recommended E%/day from fat, but urban adolescents were 6.3 times more likely to exceed this recommendation *(P < 0.01; 95% CI, 2.5-16.1)*. With regard to sodium recommendations, 85.6% participants consumed more than 1.5 g of sodium /day and 46.1% consumed more than 2.3 g of sodium/day. There was no statistical difference according to UBN or place of residence.

Only 11% of adolescents achieved the WHO-recommended fruit and vegetable intake of 400 g per day.

### Energy sources (food groups)

Energy sources per day are illustrated in Table 2. Overall, the main sources of energy (in total more than 50% of daily energy intake) in the diet were refined cereals, such as white rice, refined wheat products and processed food. In contrast, less than 1% of the daily energy intake originated from whole grain cereals. Furthermore, only 26% of the participants consumed any kind of whole grain (median intake among consumers of whole grains was 33.0 g; (25^th^-75^th^, 12.5-60.9 g). Urban adolescents consumed more red meat (*P < 0.01)* and dairy (*P < 0.01),* but less rice (*P < 0.01)* and tubers (*P < 0.01)* than their rural counterparts. With regard to socioeconomic differences, adolescents from the higher strata ate less rice (*P < 0.01)* and vegetable oil (*P = 0.02)* but more processed food (*P < 0.01)* than those in the lower strata.

**Table 2:** **Energy sources per day (median 25** ^**th**^ **-75** ^**th**^ **) in order of importance according to UBN and place of residence**

Energy sources	Overall (n = 779)		UBN ^a^						Place of residence					
			Poor		Better off		β%	***P***	Rural		Urban		β***%***	***P***
White Rice (E%)^b^	23.0	[16.2;29.6]	25.4	[19.7;32.4]	19.0	[12.8;25.9]	−22.0	<0.01	27.6	[23.0;33.7]	21.4	[14.7;28.3]	−22.1	<0.01
Processed Food^c^ (E%)	19.3	[13.4;24.8]	18.4	[12.6;23.5]	21.3	[15.3;27.1]	17.5	<0.01	17.6	[11.7;22.7]	20.1	[14.1;25.7]	6.7	0.28
Refined Wheat (E%)	10.4	[6.5;14.7]	10.3	[6.3;14.2]	10.4	[6.8;15.1]	−3.0	0.67	10.7	[5.8;14.7]	10.3	[6.7;14.7]	7.0	0.35
Dairy (E%)	7.1	[3.6;10.9]	6.3	[3;9.7.0]	8.2	[4.9;11.8]	10.5	0.11	4.6	[2.1;7.7]	7.9	[4.4;11.5]	57.5	<0.01
Red Meat (E%)	6.2	[3.5;9.7]	5.6	[3.1;9.1]	7.1	[4.2;11.0]	6.1	0.26	4.3	[2.1;7.4]	6.8	[4.0;10.5]	32.1	<0.01
Poultry (E%)	4.8	[1.5;8.5]	4.7	[1.6;9.5]	5.2	[1.6;8.5]	2.2	0.82	3.6	[1.2;7.1]	5.3	[1.7;8.7]	14.1	0.17
Vegetable Oils (E%)	3.8	[2.5;5.3]	5.4	[2.6;5.4]	3.6	[2.4;5.2]	−12	0.02	3.8	[2.4;5.0]	3.8	[2.6;5.4]	14.3	0.11
Total fruit (E%)	3.0	[1.4;3.5]	3.2	[1.5;5.7]	2.9	[1.3;5.3]	−7.6	0.31	2.9	[1.4;5.4]	3.1	[1.5;5.6]	6.2	0.52
Tubers (E%)	2.4	[1.3;4.0]	2.5	[1.5;4.2]	2.1	[1.1;3.6]	−6.9	0.43	3.1	[2.2;5.1]	2.1	[1.1;3.6]	−39.3	<0.01
Animal fat (E%)	1.2	[0.3;3.1]	1.1	[0.3;2.5]	1.3	[0.3;3.5]	24.3	0.08	1.1	[0.4;2.1]	1.2	[0.3;3.3]	11.3	0.37
Whole grain cereals (E%)	0.1	[0.0;0.2]	0.1	[0.0;0.3]	0.1	[0.0;0.2]	−27.0	0.11	0.1	[0.0;0.3]	0.1	[0;0.2.0]	23.5	0.42
Vegetables (E%)	0.7	[0.4;1.0]	0.7	[0.4;1.1]	0.6	[0.4;0.9]	−1.1	0.86	0.8	[0.5;1.2]	0.6	[0.3;1.0]	−15.3	0.18
Fish and Seafood (E%)	0.5	[0.0;3.1]	0.6	[0.0;2.8]	0.5	[0.0;3.1]	8.0	0.65	0.7	[0.0;4.3]	0.5	[0;2.6.0]	−30.9	0.20
Whole grain cereals (E%)	0.1	[0.0;0.2]	0.1	[0.0;0.3]	0.1	[0.0;0.2]	−27	0.11	0.1	[0.0;0.3]	0.1	[0;0.2.0]	23.5	0.42
Other sources^d^ (E%)	6.1	[2.4;11.2]	5.9	[2.3;11.8]	6.1	[2.4;10.4]	7.1	0.36	6.9	[3.1;15.3]	5.9	[2.3;10.5]	−11.2	0.07

^a^Unsatisfied basic needs. ^b^Energy percentage. ^c^Processed food includes sweets, snacks, soft drinks and any other packaged food item. ^d^Other sources includes: plantain, maize, other cereals, oilseeds, legumes spices and coffee.

Differences using linear regression models adjusted for gender UBN and place of residence where appropriate.

### Protective food groups

Total fruit intake was reported by 92% participants and the median total fruit intake was 121.0 g/day (25^th^-75^th^, 58.1-215.4 g). Urban adolescents were 2.6 times more likely to eat fruit than rural adolescents *(P < 0.01; 95% CI, 1.6-4.2*).Fruit was mainly consumed in three forms: (i) whole fresh fruit (58.4%), (ii) fresh fruit used in sugary juices (32.0%.) and (iii) as fresh fruit used in sugary milkshakes (4.6%). Vegetable intake was reported by 99% of the adolescents, and the median vegetable intake was 50.1 g/day (25th-75th, 24.5-81.9). Vegetables were consumed in salads (73%), as ingredients of composed recipes (22%) and in soups (5%). Legumes were consumed by 57% of the participants, its median intake was 12.7 g/day (25th-75th, 0.0-49.4), used for “menestras” (49%), soups (38%) and salads (13%).

Oilseeds were only consumed by 12% of the adolescents and the median intake among consumers was 14.5 g/day (25th-75th, 2.7-20). Urban adolescents were 5 times more likely to consume oilseeds (P < 0.01; 95% CI, 1.6-14.4) as ingredients for local typical main dishes (75%), cakes (13%) and pure oilseeds (12%).

Fish and seafood were rarely consumed: only 38% of participants reported any intake, with a median intake of 57.1 g/day (25^th^-75^th^, 28.1-87.5 g among consumers). Fried fish contributed to 44% of total fish consumption, followed by canned fish at 33.5% and fresh fish at 22.4%. Urban adolescents consumed more fresh fish and were 7.0 *(P < 0.01; 95% CI, 2.9-16.9)* times more likely to consume fresh fish than rural adolescents. In contrast, rural participants were 2.5 times more likely to eat canned fish (P < 0.01*; 95% CI, 1.3-4.7*).

### Energy sources at different mealtimes

Eighty-two-percent of the adolescents had breakfast, which constituted by refined wheat in form of bread, dairy and processed food as sugar and cocoa. The three snack times are rich in processed food. Lunch consisted of white rice, processed food in the form of sugar used for juices and milkshakes, meat, tuber, vegetable oil, fruit and vegetables. Dinner is similar to lunch but with a lower meat consumption. Urban adolescents seemed to drink more milk during breakfast and refreshments. The night refreshment was the least important meal as it was consumed by only 18% of the adolescents overall and only by 12% of the participants in the rural area.

### Dietary patterns

Two dietary patterns emerged from the factor analysis (Table 3). The first is a “rice-rich non-animal fat pattern”, which reflects a high intake of white rice, vegetable oil and tubers together with a lower contribution of animal fat, dairy products, pre-packaged food and other cereals to the diet. The second dietary pattern, a “wheat-dense animal-fat pattern”, is mainly based on refined wheat products, red meat, animal fat, dairy and plantain intake with low maize and whole grains consumption. None of the patterns appear to be “healthy” as both are rather rich in refined carbohydrates or animal sources. Moreover, food groups identified as protective factors such as fruit, vegetables, whole grains and fish were not important constituents of these patterns. Some differences in terms of nutritional content were observed between the two identified patterns as illustrated in Table 4. In the case of the “rice-rich non-animal fat pattern”, carbohydrates were a more important source of energy, mainly due to carbohydrates other than added sugar, (P < 0.01 for all) While, protein, fat energy and added sugar supply tend to decreases through the score tertiles of the “rice-rich non-animal fat pattern” (fat: P = 0.05 for rural and P < 0.01 for urban) (protein: P = 0.04 for all) (added sugar: P < 0.01 for all).. For the “wheat-dense animal-fat pattern” a lower carbohydrate (P < 0.01 for all) and fiber (P = 0.01 for rural and P < 0.01 for urban) intake is observed, together with a higher fat (P < 0.01 for all) intake (P = 0.04 for all) through the score tertiles.

**Table 3:** **Food patterns loadings using food groups’ energy percentage per day**

Food group	“Rice-rich non-animal fat pattern”	“Wheat-dense animal-fat pattern”
White Rice	**0.8**	0.0
Refined Wheat	−0.1	**0.5**
Other refined cereals	**−0.4**	−0.1
Whole grains	0.0	**−0.3**
Maize	0.1	**−0.5**
Tubers	**0.3**	0.0
Plantain	0.2	**0.4**
Legumes	0.2	−0.2
Total fruit	0.0	−0.2
Vegetables	0.2	0.0
Poultry	0.1	−0.2
Red meat	0.0	**0.4**
Fish and seafood	−0.1	−0.2
Dairy	**−0.5**	**0.3**
Oilseeds	−0.2	0.1
Vegetable oil	**0.6**	**0.4**
Animal fat	**−0.4**	**0.4**
Processed food^a^	**−0.5**	−0.2
Coffee	0.2	0.1
% Variance explained	10.8	19.0

^a^Processed food includes sweets, snacks, soft drinks and any other packaged food item.

Spices were removed as food groups as the factor loading was <0.10.

**Bold:** Factor loadings greater than 0.3.

**Table 4:** **Nutrient content (median 25** ^**th**^ **-75** ^**th**^ **) of food patterns among score tertiles**

	Population	Tetile 1		Tertile 2		Tertile 3		β%	***P***
**“Rice-non animal fat pattern**									
Energy (kcal)	Rural	1925	[1119;2117]	1516	[1332;1884]	1857	[1551;2177]	14.6	0.02
	Urban	1853	[1483;2296]	1730	[1418;2073]	2037	[1778;2406]	3.3	0.04
Carbohydrates (E%)	Overall	56.0	[ 51.6; 60.4]	61.2	[ 57.5; 65.1]	63.7	[ 60.4; 67.6]	5.6	<0.01
Other CH (E%)	Overall	37.5	[ 37.5; 41.7]	44.8	[ 41.1; 48.6]	51.9	[ 46.3; 56.2]	14.5	<0.01
Added Sugar (E%)	Overall	18.2	[ 14.4; 22.8]	15.9	[ 13.0; 19.2]	12.6	[ 9.3; 15.5]	−16.6	<0.01
Fiber (g)	Overall	11.0	[ 8.0; 15.1]	10.3	[ 7.4; 14.0]	12.4	[ 8.6; 18.2]	3.8	0.16
Total fat (E%)	Rural	25.3	[ 23.1; 27.8]	22.2	[ 19.7; 23.5]	21.4	[ 17.8; 23.9]	−7.0	0.05
	Urban	28.3	[ 24.7; 32.4]	24.6	[ 21.8; 27.6]	22.0	[ 19.8; 24.5]	−11.3	<0.01
Protein (E%)	Overall	14.2	[ 12.7; 15.8]	13.3	[ 11.8; 15.0]	12.7	[ 11.0; 14.7]	−2.9	0.04
Sodium (mg)	Rural	1728	[1175;2581]	1842	[1478;2278]	2214	[1829;2775]	20.3	0.01
	Urban	2356	[1736;3138]	2057	[1666;2697]	2422	[1960;3010]	1.6	0.45
**“Wheat-animal fat pattern”**									
Energy (kcal)	Overall	1836	[1516;2196]	1772	[1418;2124]	1919	[1585;2263]	0.8	0.53
Carbohydrates (E%)	Overall	62.2	[57.8;66.8]	60.7	[56.3;64.6]	58.5	[ 53.9; 63.0]	−2.6	<0.01
Other CH (E%)	Overall	47.1	[41.3;52.6]	44.4	[38.9;50.0]	42.2	[ 35.8; 48.1]	−3.4	<0.01
Added Sugar (E%)	Overall	15.0	[11.3;19.2]	15.7	[12.9;18.8]	15.5	[ 12.0; 19.6]	−1.2	0.58
Fiber (g)	Rural	15.9	[11.1;24.4]	10.3	[6.9;13.0]	9.9	[ 8.4; 15.7]	−23.5	0.01
	Urban	12.9	[9.3;19.0]	10.7	[7.0;14.7]	10.0	[7.6;13.2]	−13.6	<0.01
Total fat (E%)	Overall	22.4	[19.0;26.6]	24.3	[21.5;27.7]	25.8	[22.8;30.4]	6.8	<0.01
Protein (E%)	Overall	13.1	[11.3;14.9]	13.3	[11.9;15.2]	14.1	[12.0;15.7]	1.0	0.35
Sodium (mg)	Overall	2058	[1613;2745]	2106	[1640;2797]	2454	[1949;3110]	6.0	<0.01

Differences using linear regression models adjusted for UBN, place of residence and gender where appropriate. Data were stratified by rural and urban settings when interaction terms were statistically significant.

### Cardiovascular risk factors and dietary patterns

Not many significant associations were observed between the two dietary patterns and cardiovascular risk factors (Table 5). The “rice-rich non-animal fat pattern” correlated with a moderate increase in glucose blood levels among urban adolescents (*P < 0.01)*, while the “wheat-dense animal-fat pattern” was associated with an increment in blood cholesterol (*P = 0.02)* and LDL (*P = 0.04)* among rural participants.

**Table 5:** **Cardiovascular risk factors (mean ± SD) per food pattern among score tertiles**

	Population	Tertile 1	Tertile 2	Tertile 3	β %	***P***
**“Rice-non animal fat pattern**						
BMI^a^ (kg/m^2^)	Overall	20.6±3.2	20.2±3.2	20.0±2.9	−12.7	0.41
Waist circumference (cm)	Overall	71.0±8.3	69.2±8	70.3±7.7	−19.6	0.57
SBP^b^ (mmHg)	Overall	102.2±9.7	101.3±10.2	101.3±10.1	−23.6	0.58
DBP^c^ (mmHg)	Overall	62.8±8.8	62.1±8.8	62.0±8.6	−23.5	0.51
Glucose (mg/dL)	Rural	73.7±3.5	73.6±9.0	72.6±8.8	−1.5	0.41
	Urban	72.7±7.2	77.3±21.4	77.9±10.4	3.3	<0.01
Triglycerides (mg/dL)	Overall	90.9±56.7	105.5±61.6	97.5±45.9	1.4	0.80
Cholesterol (mg/dL)	Overall	144.4±27.1	150.3±28.8	145.9±38.9	−1.4	0.95
HDL^d^ (mg/dL)	Overall	52.8±12.8	50.1±12.0	47.1±11.1	−3.7	0.93
LDL^e^ (mg/dL)	Overall	73.4±22.1	79.0±24.0	79.3±36.6	−0.9	0.93
**“Wheat-animal fat pattern”**						
BMI (kg/m^2^)	Overall	20.3±3.1	20.4±3.2	20.2±3.1	−7.9	0.51
Waist circumference (cm)	Overall	70.0±7.7	70.2±8.3	70.3±8.1	1.5	0.97
SBP (mmHg)	Rural	99.9±6.5	99.3±11.1	99.0±10.2	−70.8	0.06
	Urban	102.3±9.9	101.9±9.9	102.6±9.8	17.1	0.69
DBP (mmHg)	Overall	62.4±8.7	62.0±8.6	62.5±8.9	−17.2	0.83
Glucose (mg/dL)	Overall	74.0±8.2	76.5±18.9	74.0±8.6	−0.2	0.98
Triglycerides (mg/dL)	Overall	100.0±58.6	99.4±57.1	94.7±48.6	2.2	0.58
Cholesterol (mg/dL)	Rural	137.0±45.4	152.4±33.2	155.2±33.9	3.7	0.02
	Urban	144.8±25.9	149.3±26.9	137.4±41.4	−1.9	0.20
HDL (mg/dL)	Overall	49.0±12.6	51.0±12.3	49.5±11.3	−0.3	0.86
LDL (mg/dL)	Rural	74.5±26.6	79.4±26.8	87.8±31.7	8.4	0.04
	Urban	73.3±21.9	78.9±22.8	71.6±39.2	4.3	0.17

^a^Body mass index, ^b^Systolic blood pressure, ^c^Diastolic blood pressure, ^d^High density lipoprotein, ^e^Low density lipoprotein.

Differences using linear regression models adjusted for UBN, place of residence and gender when necessary. Data were stratified by rural and urban settings when interaction terms were statistically significan.

## Discussion

To our knowledge, this is the first study presenting a comprehensive description of the diet and its relation to cardiovascular risk in adolescents from a LMIC. The diet was alarming with regard to consumption of various foods that are strongly associated with CVD. We estimated that only 11% of the participants reached the recommended fruit and vegetable intake of 400 grams per day which is lower than the intake reported in European children [28]. One question that arises is why this group of adolescents consumes such a small amount of fruit and vegetables as Ecuador is considered one of the most bio-diverse countries in the world [29] and availability of this food group does not seem not to be an issue. Focus groups performed in adolescents from Cuenca and Nabón suggested that the low fruit consumption is a consequence of a large abundance of competitive foods (junk food) and lack of fresh fruit offered in schools [30].

Similarly, whole grains were scarcely consumed, which together with the low fruit and vegetable intake is reflected in a considerably low total fiber intake. Instead, refined cereals such as white rice and refined wheat were important sources of carbohydrates. A low whole grain and fiber consumption has been also reported among US [5, 31] and Mexican children and adolescents [12]. Low whole grain intake has been strongly and positively correlated with cardiovascular risk factors, atherosclerosis and the occurrence of CVD [6], whilst energy intake from refined carbohydrates is positively correlated with the prevalence of type II diabetes [6].

Fish was also poorly consumed, a practice noted among adolescents from other countries such as US [5]. Fish consumption has been moderately linked with a protective effect against fatal coronary heart disease [7]. Specifically a cohort study suggested that the consumption of baked or boiled, but not fried fish is linked with a lower risk of death for ischemic heart disease [22]. Oilseeds were also scarcely consumed and not often in a pure form. This leads this group of adolescents at risk of death and coronary heart disease [32, 33]. In total, 2.5 million deaths worldwide were attributable to low intake of nuts and seeds in 2010 [33]. Prospective data suggest that the risk of coronary heart disease death is 8.3% lower per each 30 g of nuts consumed weekly [32].

In addition to these dietary practices associated with higher CVD risk, we found a high consumption of added sugar from fresh-fruit-juices followed by soda and bread. These findings are similar to the added sugar consumed by US children and adolescents [34], with the only difference being that in the US population soda is the main source of added sugar followed by fruit drinks and grain desserts. Worryingly, sweetened beverages intake has been widely correlated with increased energy intake, increased body weight and type II diabetes.[12, 35, 36].

Sodium intake is also high among these adolescents, but still lower than the estimates from US [37]. Considering that this nutrient tends to be underestimated using a 24 hour recall [38], the already high proportion of adolescents exceeding the recommended sodium intake is a concern. Although sodium intake was high, we did not find any association with high blood pressure, a finding previously observed among children and adolescents. There is still a debate with regard to the relationship between sodium intake and high blood pressure in young people, especially since randomized trials performed in adolescents have shown only a small effect of sodium intake on blood pressure levels [39]. This lack of relationship is probably the result of the methods used to estimate sodium intake and the short duration of randomized trails [39].

Processed food was an important source of energy among the study population, confirming previous estimates in LMICs [40]. Initially, the intake of these foods is higher in wealthy groups, but consumption in poorer populations has steadily increased since the emerging nutrition transition [40]. Our results confirm this and show how participants from the better off group got more energy from added sugar and pre-packaged food. Additionally, urban adolescents and those in the better off group had a higher energy contribution from animal sources than their rural and poor peers. Nevertheless, these differences were small and indicate how the diet in rural areas is becoming increasingly similar to that of the urban areas.

This diet low in fruit, vegetables whole grains and fiber, but rich in processed food added sugar and sodium is comparable with the diet of adolescents from other countries such as the US [5] and Mexico [12]. Nevertheless, we can identify some peculiarities, the carbohydrate intake is higher than that reported by Mexican adolescents (61 vs 52%) while protein and fat intake was lower in our sample. Carbohydrate intake is also high in neighbouring countries, i.e. Bolivia and Peru (Baya Boti Ana, unpublished data; Javier Hidalgo Miguel, unpublished data), as well as in Asian countries [41]. The main carbohydrate and daily energy source is white rice, a seed highly consumed in the Asian region as well [41]. The replacement of one serving of rice with vegetables, fruit and whole grain bread has been associated with lower ischemic heart disease mortality in the Asian populations [41], stressing the importance of promoting a balanced diet in this group of Ecuadorian adolescents.

Among rural participants we found a positive correlation between LDL and cholesterol and the “wheat-dense animal fat pattern”, a pattern of higher fat and lower fiber intake. Previous studies have described a strong correlation between fiber intake and cholesterol levels and studies conducted in animal models suggest that fiber intake increases bile acid degradation and loss, thus decreasing the amount of cholesterol available for LDL synthesis [6]. A large prospective study demonstrated associations between carotid intima media thinness and LDL blood levels during childhood, suggesting that children at the higher LDL quartiles are at higher risk of CVD [42]. Furthermore, autopsies performed in children and adolescents have also shown associations between total cholesterol and LDL levels and atherosclerosis [42].

On the other hand the “rice-rich non-animal-fat pattern” was correlated with a small increment of blood glucose among urban adolescents. Type II diabetes is the leading cause of death in the Ecuadorian population [43], although, the mean blood glucose values in the higher tertile of this pattern are within the normal ranges, a study performed in young men concluded that subjects in the higher quintiles of normal fasting blood glucose are four times more likely to develop diabetes than those in the lower quintiles [44]. We cannot identify the “rice-rich non-animal-fat pattern” as a risk factor for diabetes development, but the fact that glucose plasma level tend to increase from tertile 1 to the tertile 3 may help to identify dietary characteristics related with increments in plasma blood glucose. The predominance of refined cereals as carbohydrate sources (white rice), the poorer intake of protein and lack of whole grains maybe responsible of the higher glucose plasma level in the higher tertiles of this pattern. The poor protein content may decrease the clearance of glucose from blood [23]. In the other hand, foods rich in refined carbohydrates may displace whole grain intake [41], depriving their protective effect of reducing glucose response, and insulin sensitivity [6], furthermore, a systematic review of prospective studies concluded that the intake of two serving per day of whole grain cereals may decrease the risk of type II diabetes in 21% [45].

We have previously reported in the same population that rural adolescents are almost three times as more likely to suffer from dyslipidaemia [14]. We hypothesize that rural participants were more recently exposed to a diet poor in fiber, which may have a greater effect on their lipid profiles. The ethnic differences between the rural and urban studied population are also important. The last national demographic survey, showed how an estimated 32% of the population in Nabón was considered indigenous while this is only 1% in Cuenca [46]. In addition, the mismatch theory of early nutritional deficits followed by excesses later in life is important in LMICs [47]. It could be that rural adolescents were exposed to a higher nutritional deficiency during early childhood followed by an improvement later in life.

Although glucose levels are within normal ranges and only a small proportion of participants show high glucose blood levels [14], it seems that dietary patterns with a higher refined carbohydrate content are correlated with an increment in glucose blood levels. Meanwhile diets richer in fat and lower in fiber are positively correlated with LDL and cholesterol blood concentrations.

We acknowledge some limitations in this study. First, we could not compile information on saturated fat content of all the foods consumed. This is unfortunate as the type of fat is vital to the reduction of LDL cholesterol and concomitant reduction in risk of coronary heart disease [48]. Secondly, the cross sectional nature of this study only allows for associations and cannot infer causality.

## Conclusions

The Ecuadorian adolescent population presents a number of dietary practices conducive to CVD development. Effective preventive strategies are needed to prevent CVD and other NCD in the Ecuadorian population by encouraging a balanced diet by decreasing refined cereals, soda, processed food and sodium consumption as well as increasing fruit, vegetables, whole grain cereals and nuts intake.

## Abbreviations

CVD: Cardiovascular diseases
LMICs: Low-and middle-income countries
NCD: Non-communicable diseases
USDA: United States Department of Agriculture
INNSZ: Instituto Nacional de Ciencias Médicas y Nutrición Salvador Zubirán
INCAP/OPS: Instituto de Nutrición de Centro América y Panamá /Organización Panamericana de la Salud
CENAN/INS: Centro Nacional de Alimentación y Nutrición / Instituto Nacional de Salud
DRIs: Dietary reference intakes
BMI: Body mass index
SBP: Systolic blood pressure
DBP: Diastolic blood pressure
COLT: Total cholesterol
TG: Triglycerides
HDL: High-density lipoprotein cholesterol
LDL: Low-density lipoprotein cholesterol
UBN: Unsatisfied basic needs
SD: Standard deviation
OR: Odds ratio
CI: 95% confidence interval.
